# Tunnel-Like Myocardial Structure Formed by Sequential Left Atrial Appendage Closure and Resection as a Substrate for Macroreentrant Atrial Tachycardia

**DOI:** 10.7759/cureus.103390

**Published:** 2026-02-10

**Authors:** Noeru Shibayama, Yosuke Miwa, Nobuyuki Kuroiwa, Toshiya Ohtsuka, Go Watanabe

**Affiliations:** 1 Cardiology, NewHeart Watanabe Institute, Tokyo, JPN; 2 Cardiology, Miwa Heart Clinic, Tokyo, JPN; 3 Cardiac Surgery, NewHeart Watanabe Institute, Tokyo, JPN

**Keywords:** atrial tachycardia, catheter ablation, left atrial appendage closure, left atrial appendage resection, macroreentrant tachycardia, mitral valve surgery, slow conduction pathway, surgical arrhythmogenic substrate, tunnel-like myocardial structure

## Abstract

Left atrial appendage closure (LAAC) is frequently performed during cardiac surgery to reduce thromboembolic risk; however, incomplete closure with residual appendage flow is not uncommon. In selected cases, additional left atrial appendage resection (LAAR) may be undertaken, although the electrophysiological consequences of this sequential surgical strategy remain poorly characterized. We report a 59-year-old man with a history of mitral valve repair, tricuspid annuloplasty, Cox-Maze procedure, and surgical LAAC who presented with sustained atrial tachycardia (AT). Persistent residual appendage flow following LAAC necessitated subsequent stapler-based LAAR. Postoperative contrast-enhanced computed tomography revealed a contrast-filled, tunnel-like myocardial structure bridging the LAAC site and the LAAR plane, remaining endocardially continuous with the left atrium and adjacent to the mitral annulus. High-density electroanatomic mapping demonstrated macroreentrant AT anatomically and functionally associated with this surgically created myocardial structure, supported by entrainment pacing findings. Radiofrequency ablation at sites anatomically adjacent to the tunnel-like structure resulted in reproducible prolongation of the tachycardia cycle length, with definitive termination achieved only after additional perimitral and pericoronary sinus ablation. This case highlights a previously unrecognized postoperative arrhythmogenic substrate created by sequential LAAC and LAAR. It underscores the importance of integrating preprocedural imaging with detailed electrophysiological mapping to identify noncanonical conduction pathways in patients with complex surgically altered atrial anatomy.

## Introduction

Concomitant left atrial appendage closure (LAAC) during cardiac surgery reduces the risk of stroke in patients undergoing procedures such as mitral valve repair or coronary artery bypass grafting [1]. However, incomplete surgical closure with residual flow or stumps is common, occurring in approximately one-quarter of patients in contemporary imaging-based studies [2-3]. Such residual leaks may impair the effectiveness of LAAC in preventing left atrial appendage-related thrombosis and are associated with an increased risk of thromboembolic events [3]. Although anticoagulation therapy is often considered in the presence of residual leaks, standardized guidelines for the management of residual leaks after LAAC are lacking, and optimal management strategies remain poorly defined, particularly in patients with unreliable anticoagulation adherence [4]. In selected cases, additional surgical intervention such as left atrial appendage resection (LAAR) may be performed to eliminate residual appendage flow. The anatomical configuration resulting from sequential LAAC followed by LAAR can vary depending on the orientation and spatial relationship of the closure and resection lines. In rare circumstances, this sequence may enclose residual atrial myocardium between the two surgical sites. Although no longer part of the functional appendage, such tissue may remain electrically viable and endocardially continuous with the left atrium, forming a tunnel-like myocardial structure that may serve as an arrhythmogenic substrate for atrial arrhythmias [5]. Here, we report a case in which sequential LAAC and LAAR resulted in the formation of a tunnel-like myocardial structure that was functionally associated with macroreentrant atrial tachycardia (AT). While atrial tachyarrhythmias following mitral valve surgery or LAAC have been described [6-7], macroreentrant AT potentially involving a surgically created tunnel-like myocardial structure formed by sequential LAAC and LAAR has not, to our knowledge, been previously reported. This case highlights a previously unrecognized arrhythmogenic substrate created by sequential left atrial interventions and underscores the potential electrophysiological consequences of complex surgical appendage management.

## Case presentation

**Figure 1:**
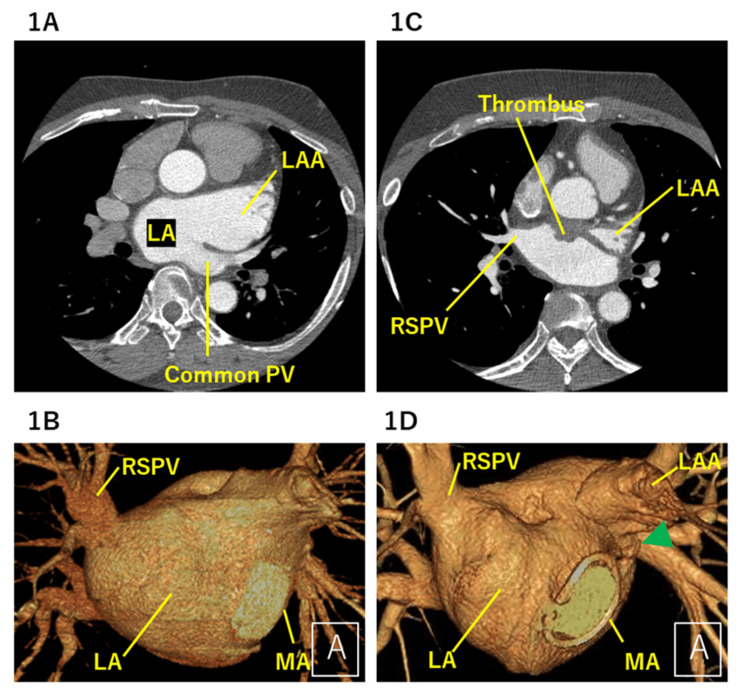
Contrast-enhanced CT before and after surgical LAAC (A) Axial contrast-enhanced CT image obtained before cardiac surgery demonstrating a markedly enlarged LA, LAA, and a left common pulmonary vein. (B) Three-dimensional CT reconstruction preoperatively showing the anatomical relationships among the LA, LAA, mitral annulus, and pulmonary veins. (C) Axial contrast-enhanced CT image obtained approximately one year after surgical LAAC, demonstrating contrast opacification within the LAA and a mural filling defect along the anterior left atrial wall adjacent to the closure site. (D) Three-dimensional CT reconstruction after LAAC demonstrates continuity between the closure site and the remnant LAA, as well as the spatial relationship to adjacent left atrial structures. CT: computed tomography, LA: left atrium, LAA: left atrial appendage, LAAC: left atrial appendage closure, PV: pulmonary vein, RSPV: right superior pulmonary vein, MA: mitral annulus

A 59-year-old man presented with exertional dyspnoea. Three years before the presentation, he underwent mitral valve repair and tricuspid annuloplasty for valvular regurgitation. During the same operation, a Cox-Maze procedure using cryoablation and a suture-based LAAC was performed for persistent atrial fibrillation. Preoperative contrast-enhanced computed tomography (CT) demonstrated a markedly enlarged left atrial appendage and a left common pulmonary vein (Figure 1A-1B).

Approximately one year after surgery, follow-up contrast-enhanced CT revealed persistent blood flow within the left atrial appendage and a mural thrombus located along the anterior wall of the left atrium, adjacent to the incomplete LAAC site (Figure 1C, 1D). At that time, the patient reported poor adherence to anticoagulation therapy. Direct oral anticoagulant therapy with apixaban (5 mg twice daily) was initiated; however, follow-up CT performed several months later demonstrated persistent thrombus. Under close monitoring of anticoagulation adherence, subsequent imaging eventually demonstrated radiographic resolution of the thrombus, although residual appendage flow persisted.

Given the persistent residual leak and ongoing thromboembolic risk, a multidisciplinary discussion was held. Approximately two years after the initial surgery, the patient underwent stapler-based LAAR. At the same time, epicardial radiofrequency ablation was performed along the left pulmonary veins.

**Figure 2:**
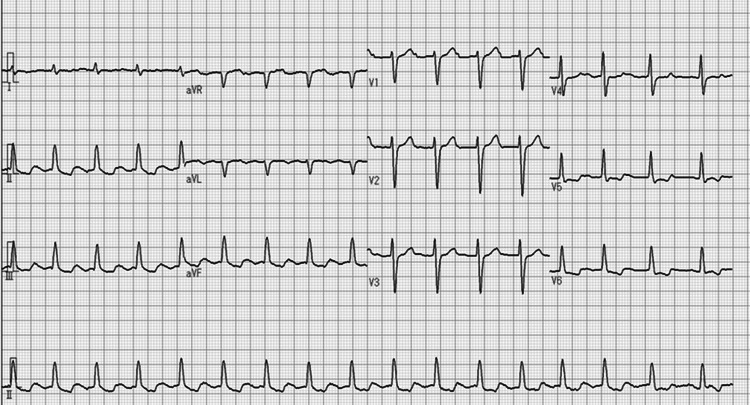
Surface electrocardiogram Twelve-lead surface electrocardiogram demonstrating a regular AT with a cycle length corresponding to a heart rate of approximately 102 beats per minute. AT: atrial tachycardia

Despite these interventions, AT persisted and gradually became symptomatic, with the development of exertional dyspnoea. Although AT had been present since the initial mitral valve surgery, worsening symptoms prompted referral to the cardiology department, and catheter ablation was planned. Twelve-lead electrocardiography demonstrated organized AT with a heart rate of approximately 102 beats per minute (Figure 2).

Transthoracic echocardiography showed a left ventricular ejection fraction of 45%, left ventricular diastolic and systolic dimensions of 51 and 41 mm, respectively, a left atrial diameter of 50.8 mm, and a left atrial volume index of 40 mL/m².

**Figure 3:**
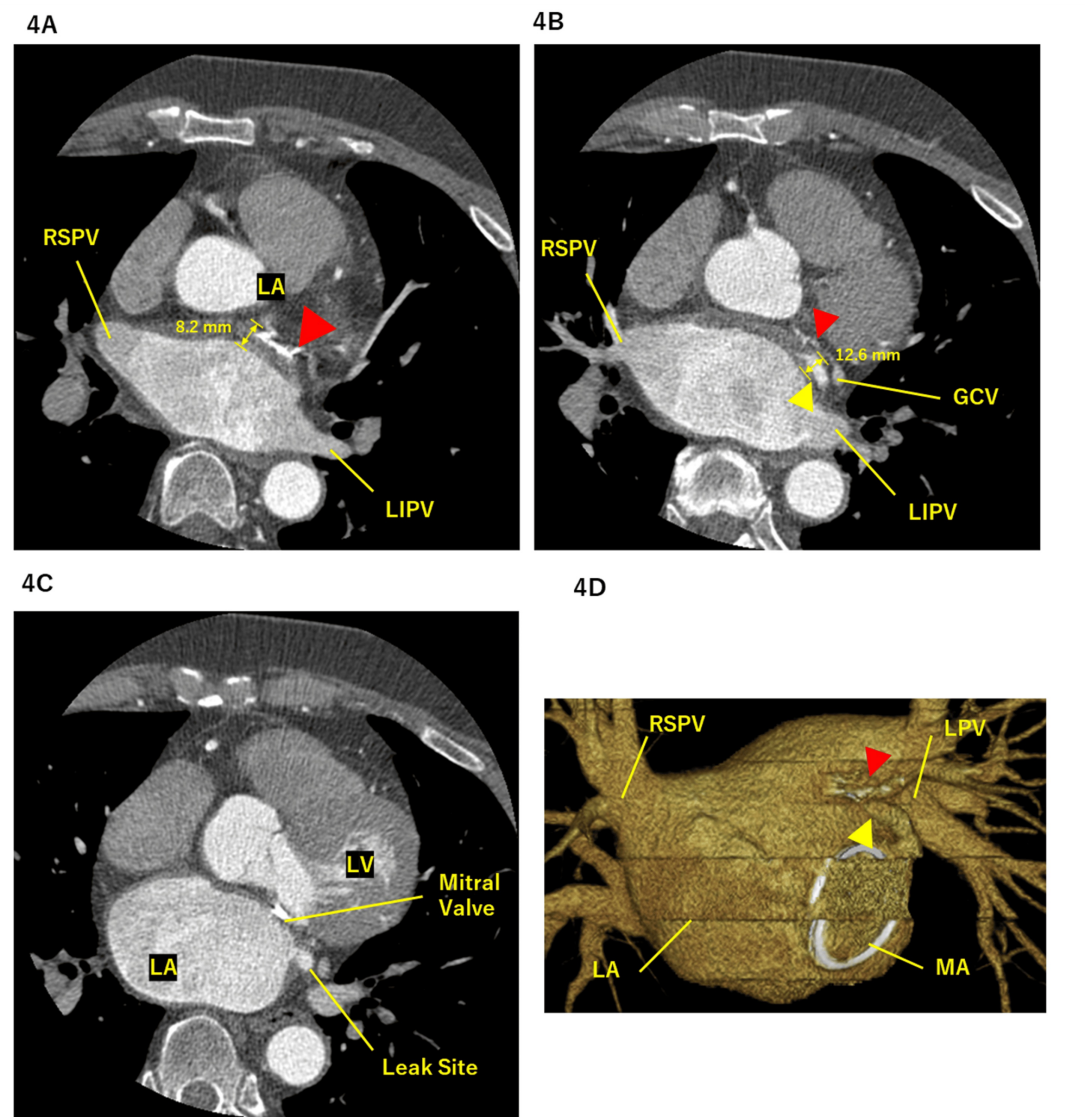
CT and three-dimensional reconstruction demonstrating a tunnel-like myocardial structure formed between the LAAC and LAAR sites (A) Axial contrast-enhanced CT image after LAAC demonstrating the LA, pulmonary veins, and residual contrast filling adjacent to the prior closure site. (B) Axial contrast-enhanced CT image demonstrating a contrast-filled myocardial channel extending between the LAAC site and the subsequent LAAR plane, corresponding to a tunnel-like myocardial structure (yellow arrowhead). (C) Axial contrast-enhanced CT image demonstrating the residual leak site communicating with the left atrial cavity adjacent to the lateral LA wall and MA. (D) Three-dimensional volume-rendered CT reconstruction illustrating the tunnel-like myocardial structure (yellow arrowhead) bridging the LAAC site and the LAAR plane, as well as the stapler used for LAAR (red arrowhead). CT: computed tomography, LA: left atrium, LPV: left pulmonary vein, LIPV: left inferior pulmonary vein, RSPV: right superior pulmonary vein, MA: mitral annulus, LV: left ventricle, GCV: great cardiac vein, LAAC: left atrial appendage closure, LAAR: left atrial appendage resection

Several months later, preprocedural contrast-enhanced CT was performed, which demonstrated no residual mural thrombus and revealed a contrast-filled myocardial channel extending from the prior LAAC site to the stapler-based LAAR plane, adjacent to the mitral annulus (Figure 3A-3D).

**Figure 4:**
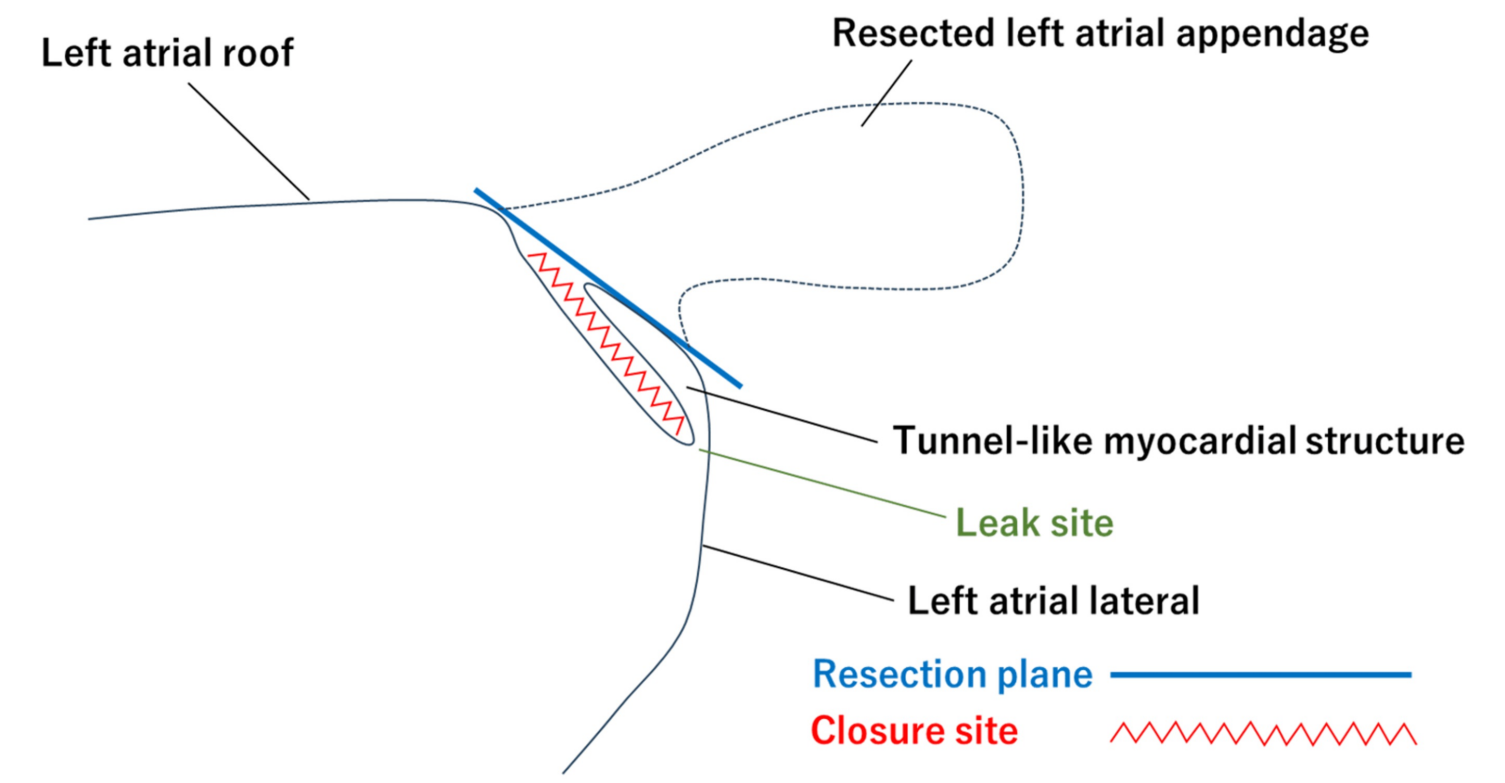
Schematic illustration of the tunnel-like myocardial structure Schematic illustration of a tunnel-like myocardial structure formed by sequential LAAC and LAAR. The red zigzag line marks the closure site, and the blue line indicates the resection plane. The enclosed myocardial tissue forms a residual tunnel with an inferior leak site (green). LAAC: left atrial appendage closure

The channel remained in communication with the left atrial cavity, and three-dimensional reconstruction revealed a continuous myocardial bridge between the closure and resection lines, with continuity to the anterior left atrial wall rather than an isolated appendage remnant. To clarify this anatomical configuration, a schematic illustration was created depicting residual atrial myocardium enclosed between the LAAC site and the LAAR plane, forming an endocardially continuous tunnel-like myocardial structure (Figure 4).

The wall thickness of this structure measured 8.2-12.6 mm, exceeding reported normal left atrial wall thickness values, which generally range from approximately 1 to 7 mm depending on the atrial region [8].

**Figure 5:**
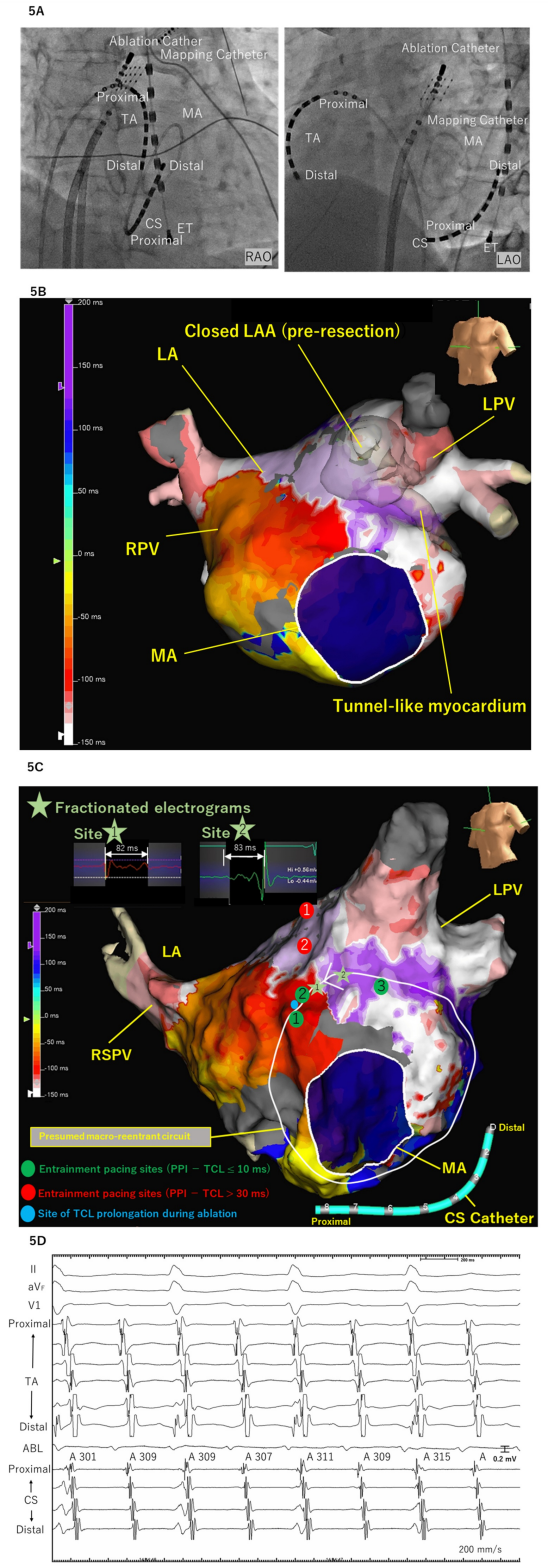
Electrophysiological findings demonstrating macroreentrant AT anatomically associated with the tunnel-like myocardial structure (A) Fluoroscopic images in the RAO and LAO projections showing catheter positions during the electrophysiological study. The ablation catheter, high-density mapping catheter, and CS catheter are positioned in the LA, MA, and CS, respectively. (B) Three-dimensional electroanatomic activation map of the left atrium during AT, integrated with contrast-enhanced CT, demonstrating a macroreentrant activation pattern propagating around the perimitral region. The reentrant wavefront courses anatomically adjacent to the tunnel-like myocardial structure (outlined). (C) Electroanatomic map illustrating the presumed macroreentrant circuit. Green dots indicate entrainment pacing sites along the adjacent left atrial myocardium with near-equal PPI−TCL values (Site 1 = +3 ms, Site 2 = +4 ms, Site 3 = +7 ms). Red dots indicate entrainment pacing sites with PPI−TCL > 30 ms, indicating exclusion from the reentrant circuit (Site 1 = 190 ms; Site 2 = 170 ms). Light green stars (Sites 1 and 2) indicate locations where fractionated electrograms with prolonged durations (82 ms and 83 ms, respectively) were recorded. The blue dot denotes the site of initial TCL prolongation during radiofrequency ablation. (D) Intracardiac electrograms recorded during radiofrequency ablation at a site along the anterior left atrial wall anatomically adjacent to the tunnel-like myocardial structure, demonstrating progressive prolongation of the AT cycle length from 301 ms to 315 ms. TA: tricuspid annulus, MA: mitral annulus, CS: coronary sinus, ET: esophageal temperature, RAO: right anterior oblique, LAO: left anterior oblique, TCL: tachycardia cycle length, PPI: post-pacing interval, AT: atrial tachycardia, CT: computed tomography

An electrophysiological study was performed during sustained AT. A BeeAT catheter (Japan Lifeline, Tokyo, Japan) was positioned in the coronary sinus and tricuspid annulus, an Advisor HD Grid catheter (Abbott, Abbott Park, IL, USA) was used for high-density left atrial mapping, and an irrigated ablation catheter (TactiFlex, Abbott) was advanced via a steerable sheath (Agilis, Abbott). Three-dimensional electroanatomic mapping was performed using the EnSite X system (Abbott) (Figure 5A).

Activation mapping demonstrated conduction delay and fractionated electrograms along the anterior left atrial wall. When integrated with contrast-enhanced CT, the reentrant activation pattern was anatomically adjacent to the previously identified tunnel-like myocardial structure (Figure 5B).

Activation mapping demonstrated a presumed macroreentrant circuit originating from the region adjacent to the LAAR site. Activation propagated inferiorly along the anterior left atrial wall, traversed the perimitral region, ascended along the lateral left atrium, and returned toward the anterior left atrium along a pathway anatomically adjacent to the tunnel-like myocardial structure (Figure 5C). The total tachycardia cycle length (TCL) was 301 ms. Entrainment pacing revealed post-pacing interval minus TCL (PPI−TCL) values of −2 ms at the proximal coronary sinus, +7 ms at the distal coronary sinus, and +170 ms at the distal tricuspid annulus, indicating perimitral participation and exclusion of the tricuspid annulus from the reentrant circuit. Entrainment pacing was additionally performed at two sites along the left atrial roof, both of which demonstrated PPI values substantially longer than the TCL, indicating exclusion of these regions from the reentrant circuit. Entrainment pacing performed at three sites anatomically adjacent to the tunnel-like myocardial structure demonstrated PPI−TCL values of +3 ms, +4 ms, and +7 ms, respectively, all with concealed fusion (Figure 5C). Fractionated electrograms recorded at two sites near the resection plane demonstrated prolonged durations of 82 ms and 83 ms, accounting for approximately 27% of the TCL.

Radiofrequency ablation was delivered endocardially using an irrigated catheter at 40 W with dragging applications guided by local electrogram characteristics. Lesions were applied along the anterior left atrial wall and the stapled ridge of the resected left atrial appendage. No steam pops or impedance abnormalities occurred during ablation.

**Figure 6:**
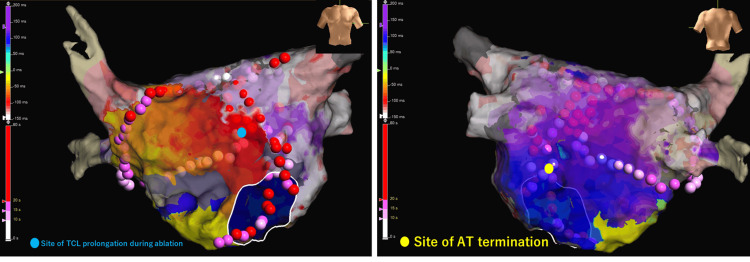
Final ablation lesion sets and sites of tachycardia modification and termination during catheter ablation Electroanatomic maps of the left atrium showing the final radiofrequency ablation lesion sets during AT ablation. Red, pink, and white dots represent delivered ablation lesions along the anterior left atrial wall, perimitral region, and pericoronary sinus region. The blue dot indicates the site where initial TCL prolongation was observed during ablation. The yellow dot denotes the site at which AT termination was achieved during endocardial ablation in the pericoronary sinus region following mitral isthmus modification. AT: atrial tachycardia, TCL: tachycardia cycle length

Ablation delivered at a site along the anterior left atrial wall, adjacent to the previously identified tunnel-like myocardial structure, resulted in gradual prolongation of the TCL from 301 ms to 315 ms (Figure 5D). However, tachycardia termination was achieved only after additional endocardial ablation along the pericoronary sinus region following mitral isthmus ablation. Following termination, box isolation of the pulmonary veins and posterior left atrial wall was completed (Figure 6).

No atrial tachyarrhythmias were inducible. The patient remained asymptomatic and free from recurrent atrial tachyarrhythmia during 28 months of follow-up.

## Discussion

This case illustrates that sequential LAAC followed by LAAR can result in the formation of a surgically created tunnel-like myocardial structure that remains endocardially continuous with the left atrium. In the present patient, AT exhibited electrophysiological characteristics consistent with perimitral macroreentry, a well-recognized arrhythmia following mitral valve surgery and Cox-Maze procedures [6]. Conduction delay and fractionated electrograms along the anterior left atrial wall are common in this setting and are not, in isolation, specific. Accordingly, the arrhythmia observed in this patient shares features with typical postoperative perimitral AT.

However, several observations suggest that the surgically created tunnel-like myocardial structure may have contributed to the reentrant substrate. Entrainment pacing performed at multiple sites anatomically adjacent to this structure demonstrated near-equal post-pacing interval minus TCL values with concealed fusion, while entrainment pacing at other left atrial regions, including the roof, excluded those areas from the circuit. In addition, fractionated electrograms recorded near the resection plane accounted for approximately 27% of the TCL, consistent with localized slow conduction. Radiofrequency ablation at sites along the anterior left atrial wall adjacent to the tunnel-like structure reproducibly prolonged the TCL, suggesting partial modification of a functionally relevant conduction pathway.

Importantly, tachycardia termination required additional ablation along the pericoronary sinus region following mitral isthmus ablation, indicating that the reentrant circuit was not confined to a single discrete isthmus. Rather, the findings support a composite perimitral reentrant mechanism involving interconnected endocardial pathways. Alternative perimitral conduction routes, including epicardial conduction via the ligament of Marshall or Bachmann bundle, cannot be excluded [6,7].

Several limitations warrant explicit acknowledgment. The ablation catheter was not confirmed to be positioned within the lumen of the tunnel-like structure using intracardiac echocardiography, and tachycardia termination was not achieved by ablation at that site alone. Therefore, this case does not provide definitive proof that the slow-conduction pathway resided within the tunnel-like structure itself. Instead, the available data support an anatomical and functional association between this surgically created structure and the reentrant circuit.

Anatomical characteristics of the tunnel-like structure may further explain its arrhythmogenic potential and relative resistance to ablation. Postoperative contrast-enhanced CT demonstrated marked wall thickening (8.2-12.6 mm), exceeding reported normal left atrial wall thickness values [8]. Increased myocardial thickness is a recognized limitation to effective lesion formation during radiofrequency ablation, and lesion depth has been shown to reach a plateau early during RF energy delivery [9].

This case highlights the importance of recognizing surgically induced anatomical substrates following complex sequential left atrial interventions. Careful integration of imaging with detailed electrophysiological mapping is essential to identify noncanonical conduction pathways and to guide effective ablation strategies in postoperative atrial tachyarrhythmias.

## Conclusions

Sequential LAAC followed by LAAR may result in the formation of an endocardially continuous tunnel-like myocardial structure that is anatomically and functionally associated with macroreentrant AT. In the present case, electrophysiological findings suggested that this surgically created structure was closely associated with the perimitral reentrant circuit. However, definitive localization of the slow-conduction pathway within the tunnel-like structure could not be established. Catheter ablation targeting regions anatomically adjacent to the tunnel-like myocardial structure resulted in reproducible modification of the tachycardia, with definitive termination achieved only after additional perimitral and pericoronary sinus ablation. This case underscores the importance of careful preprocedural imaging and detailed electrophysiological mapping in patients with complex surgically altered atrial anatomy. Reporting similar cases may help clinicians recognize uncommon postoperative substrates and refine diagnostic and ablation strategies for atrial tachyarrhythmias.
